# Dorsal vein tear during radical total penectomy

**DOI:** 10.4103/0970-1591.57909

**Published:** 2009

**Authors:** B. Satheesan, N. Kathiresan, K. T. Siddhappa

**Affiliations:** Division of Genitourinary Oncology, Cancer Institute (W.I.A.) Sardar Patel Road, Adyar, Chennai - 600 020, India; 1 Department of Surgical Oncology, Cancer Institute (W.I.A.) Sardar Patel Road, Adyar, Chennai - 600 020, India

Dear Editor,

Radical surgery for penile cancers depends on the extent of the primary tumor and the available penile stump after radical margin. Total penectomy is reserved for malignant tumors involving shaft of penis and when the residual stump is insufficient for projectile micturition. Radical total penectomy (the entire corpora cavernosa is resected) may be indicated in multifocal disease in the corpora cavernosa and deep-seated sarcomas of the penis.[1] Isolation and ligation of dorsal vein is an important step in this extirpative surgery. Injury to the dorsal vein during isolation is a rare complication. This could be troublesome if the vein is torn at the inferior margin of pubic symphysis as control is difficult. Recently, we encountered a similar problem during radical total penectomy for leiomyosarcoma of the penis. This was the first time we had come across such an unpleasant situation in our surgical practice. We hope this may be of some worth for younger surgeons who may encounter similar situation.

A 39-year-old gentleman presented with a nodular solid lesion on the glans penis of size 3 × 3 cm, which extended to the corona glandis. The wedge biopsy revealed spindle cell sarcoma grade II for which immunohistochemistry confirmed leiomyosarcoma. Staging evaluation confirmed the non-metastatic status. He was taken up for radical total penectomy. During the procedure, after the division of the suspensory ligament of the penis, the dorsal vein of the penis was dissected. But, an inadvertent injury to the lateral wall of the vein resulted in a longitudinal tear leading to significant blood loss. Attempts to secure the vessel with hemostat further extended the tear beneath the pubic arch. The distal part of the vein was controlled with the hemostat but the proximal part continued to bleed profusely. The hemorrhage was temporarily controlled with compression. An infraumbulical midline incision was made in the lower abdomen to enter the retropubic space of Retzius. The bladder was dissected down and the prostatic apex was reached after dividing the puboprostatic ligament. This brought the dorsal vein into vision and suture ligation of the same was contemplated. Despite the effort, the bleeding continued, but to a lesser extent. Hence, two options were considered. First was digital pushing of the soft tissue beneath and behind the pubic arch to facilitate the suture ligation of the vein from the pre-pubic area. If this failed, the next option was symphysiectomy and direct ligation under vision. Fortunately, the digital caudal push could tent the bleeding vein downwards, which was controlled with sutures. The blood loss during the procedure due to the tear was 1200 ml, requiring blood transfusion! Post-operatively, he had an uneventful recovery and was discharged on day 7.

It is important to avoid such injuries during this procedure as the blood loss is quite significant. Panic attempts to control bleeding by applying hemostats blindly may worsen the situation. After control of the distal part, compression would help to control bleeding till definitive a measure is pursued. Familiarity of anatomy and quick reaction holds the key to success of homeostasis in such situations.

## References

[1] Sharp DS, Angermeier KW, Wein AJ, Kavoussi LR, Novick AC, Partin AW, Peters CA (1998). Surgery of penile and urethral carcinoma. Campbell-Walsh Urology.

